# Supplementary material for: High Cell Selectivity and Bactericidal Mechanism of Symmetric Peptides Centered on d-Pro–Gly Pairs
Source: Int J Mol Sci. 2020 Feb 8;21(3):1140. doi: 10.3390/ijms21031140 (PMC7037546; doi:10.3390/ijms21031140)
Supplement: Supplementary file 1 [file ijms-21-01140-s001.pdf]

Supplementary Materials

Title:

High Cell Selectivity and Bactericidal Mechanism of Symmetric Peptides Centered on D-Pro–Gly Pairs

Authors:

Bo-yan Jia, Yi-ming Wang, Ying Zhang, Zi Wang, Xue Wang, Inam Muhammad, Ling-cong Kong, Zhi-hua Pei, Hong-xia Ma * and Xiu-yun Jiang *

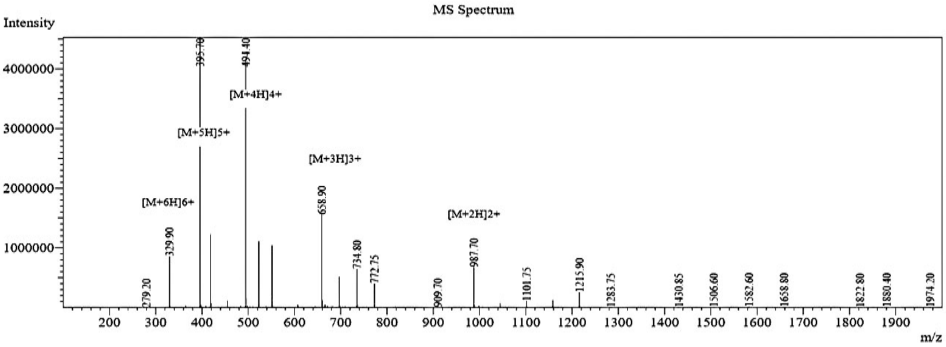

(**a**)

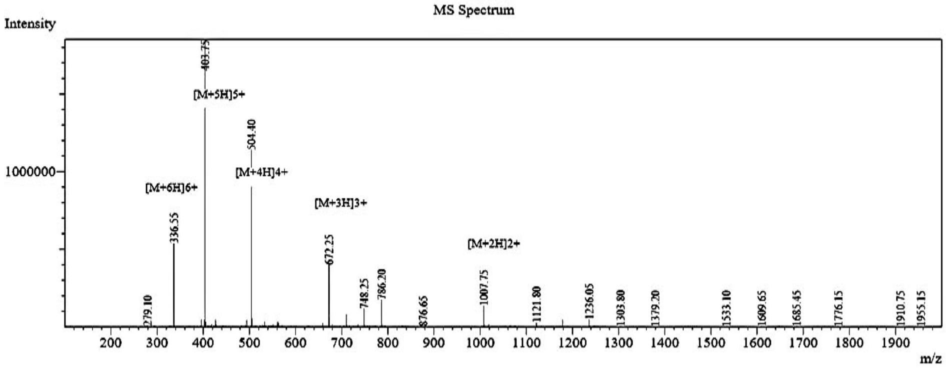

(**b**)

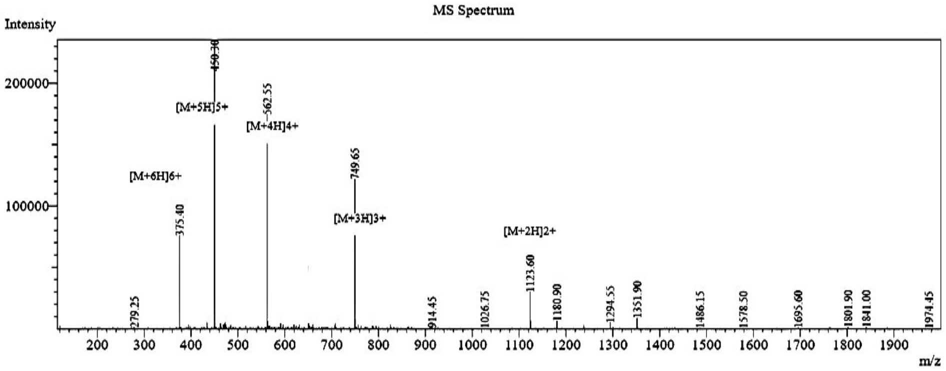

(**c**)

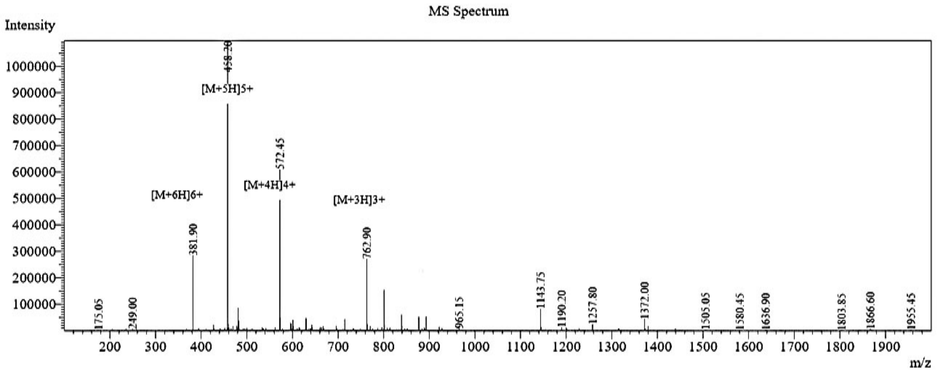

(**d**)

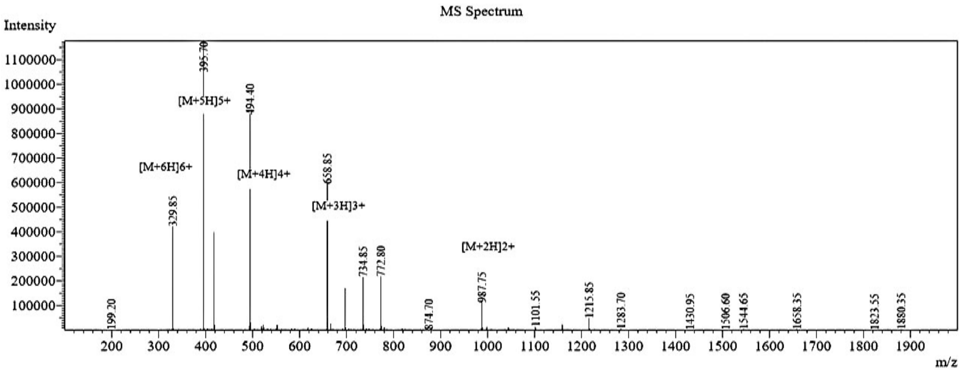

(**e**)

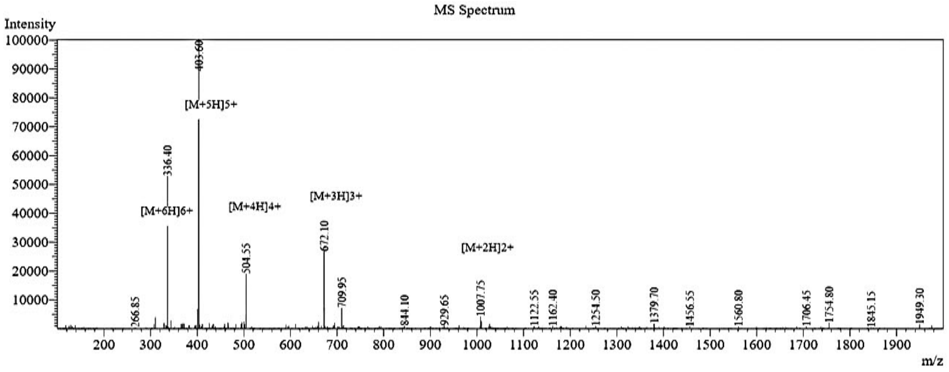

(**f**)

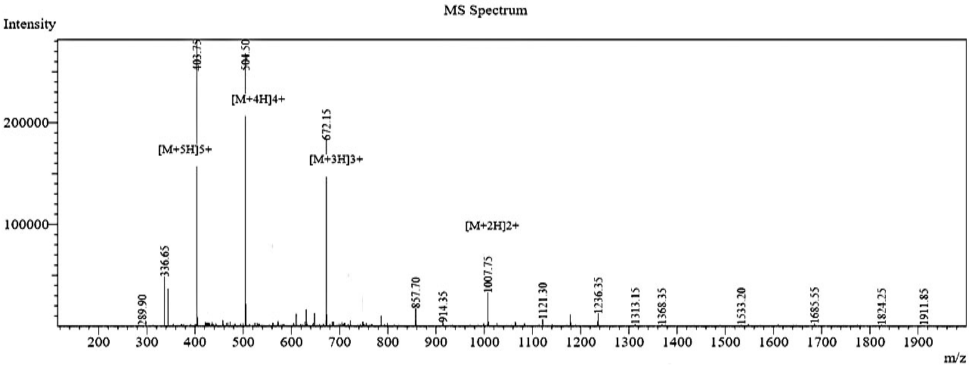

(**g**)

Figure S1. The MALDI-TOF MS of IR_{GG} (**a**), IR_{pG} (**b**), FR_{GG} (**c**), FR_{pG} (**d**), LR_{GG} (**e**), LR_{pG} (**f**) and $LR\alpha$ (**g**).

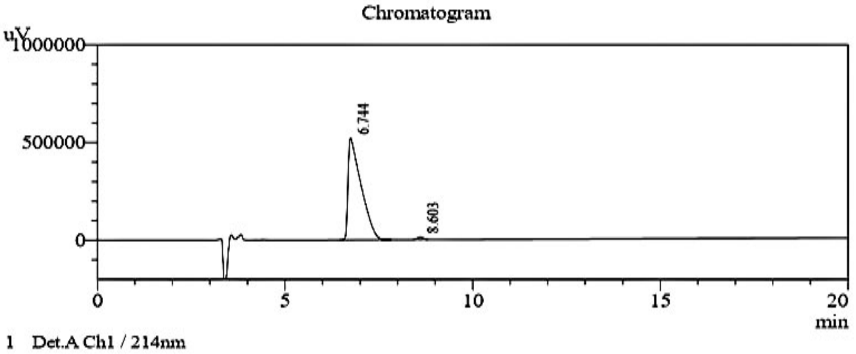

PeakTable

Detector A Ch1 214nm

Peak#	Ret. Time	Area	Height	Area %	Height %
1	6.744	12625051	521779	98.943	97.631
2	8.603	134844	12662	1.057	2.369
Total		12759894	534441	100.000	100.000

(**a**)

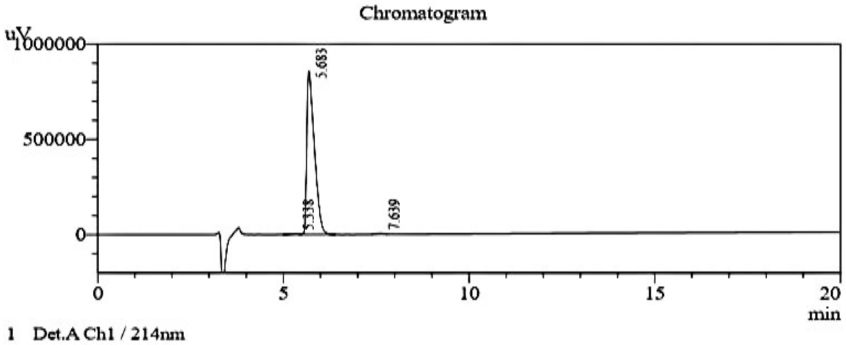

PeakTable

Detector A Ch1 214nm

Peak#	Ret. Time	Area	Height	Area %	Height %
1	5.338	71453	5384	0.577	0.620
2	5.683	12251155	859064	98.848	98.867
3	7.639	71307	4461	0.575	0.513
Total		12393915	868909	100.000	100.000

(**b**)

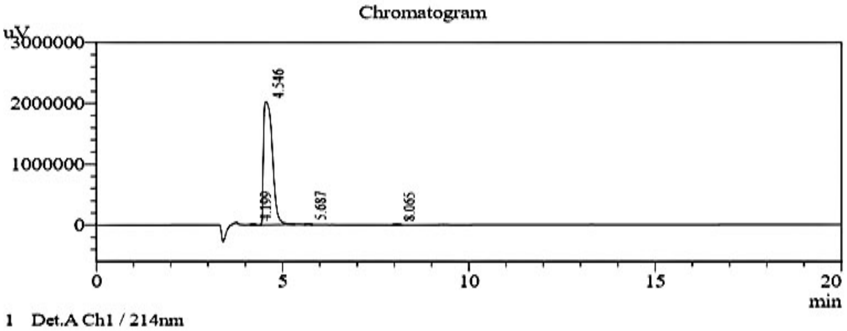

PeakTable

Detector A Ch1 214nm

Peak#	Ret. Time	Area	Height	Area %	Height %
1	4.199	86181	17176	0.256	0.833
2	4.546	33441691	2025416	99.356	98.225
3	5.687	87508	13702	0.260	0.664
4	8.065	43064	5715	0.128	0.277
Total		33658444	2062009	100.000	100.000

(**c**)

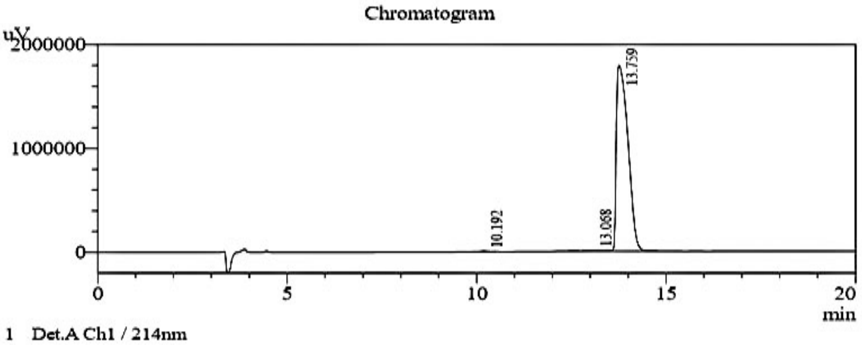

1 Det.A Ch1 / 214nm

PeakTable

Detector A Ch1 214nm

Peak#	Ret. Time	Area	Height	Area %	Height %
1	10.192	73083	7429	0.190	0.411
2	13.068	237994	10652	0.618	0.589
3	13.759	38185101	1790524	99.192	99.000
Total		38496177	1808605	100.000	100.000

(**d**)

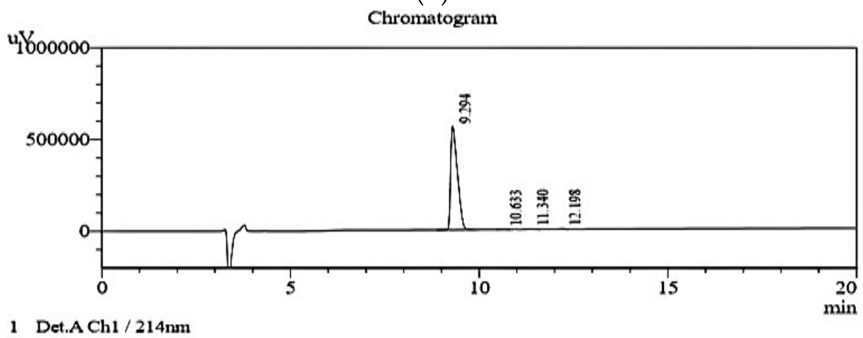

1 Det.A Ch1 / 214nm

PeakTable

Detector A Ch1 214nm

Peak#	Ret. Time	Area	Height	Area %	Height %
1	9.294	7005832	564679	99.045	98.718
2	10.633	15032	1699	0.213	0.297
3	11.340	32329	2858	0.457	0.500
4	12.198	20208	2777	0.286	0.485
Total		7073400	572014	100.000	100.000

(**e**)

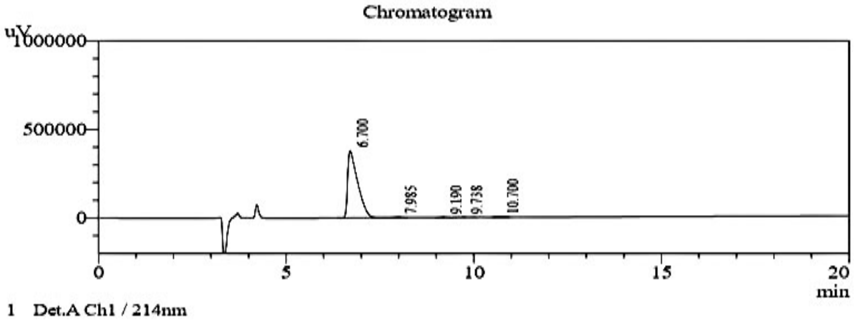

1 Det.A Ch1 / 214nm

PeakTable

Detector A Ch1 214nm

Peak#	Ret. Time	Area	Height	Area %	Height %
1	6.700	7041113	380803	98.610	98.042
2	7.985	50078	3450	0.701	0.888
3	9.190	16651	1769	0.233	0.455
4	9.738	14363	1209	0.201	0.311
5	10.700	18128	1175	0.254	0.303
Total		7140334	388406	100.000	100.000

(**f**)

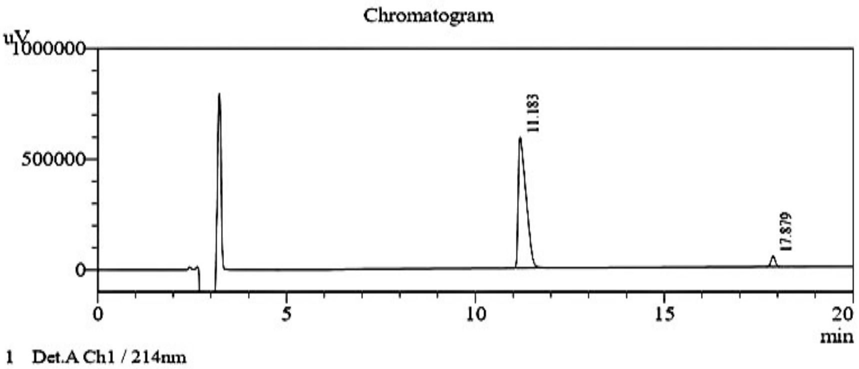

1 Det.A Ch1 / 214nm

PeakTable

Detector A Ch1 214nm

Peak#	Ret. Time	Area	Height	Area %	Height %
1	11.183	8448391	590476	95.785	92.463
2	17.879	371751	48130	4.215	7.537
Total		8820142	638606	100.000	100.000

(**g**)

Figure S2. The HPLC spectra of IR$_{GG}$ (**a**), IR$_{pG}$ (**b**), FR$_{GG}$ (**c**), FR$_{pG}$ (**d**), LR$_{GG}$ (**e**), LR$_{pG}$ (**f**) and LR*α* (**g**).

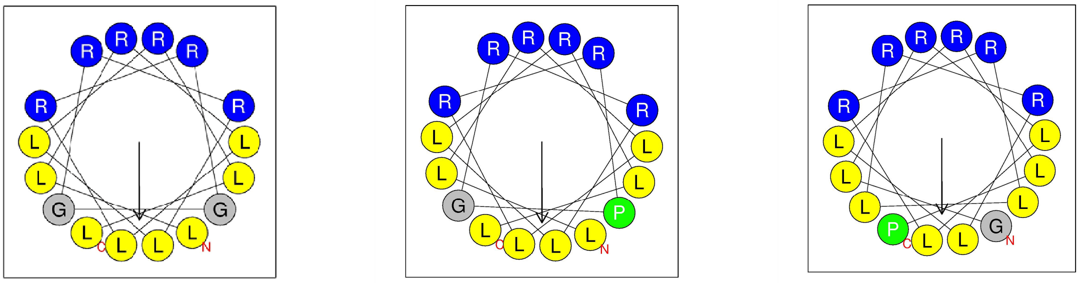

(**a**) (**b**) (**c**)

Figure S3. The helical wheel projections of LR$_{GG}$ (**a**), LR$_{pG}$ (**b**) and LR*α* (**c**). The blue represents positively charged residues. The yellow represents hydrophobicity residues. The gray and green represent glycine and proline, respectively.

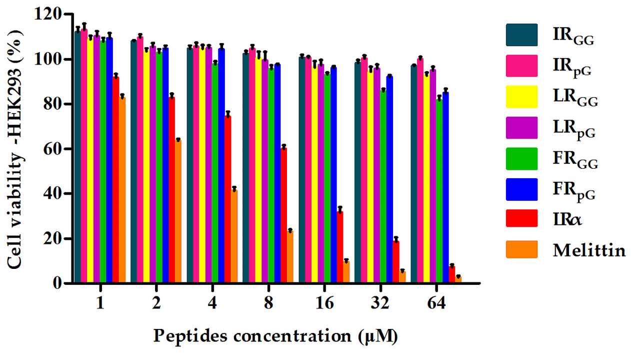

Figure S4. Cytotoxicity of the designed peptides against HEK293T cells.
